# Association of Ki-67 With Clinicopathological Factors in Breast Cancer

**DOI:** 10.7759/cureus.15621

**Published:** 2021-06-13

**Authors:** Nishit ., Jitendra S Nigam, Tarun Kumar, Shreekant Bharti, Surabhi ., Ruchi Sinha, Punam P Bhadani

**Affiliations:** 1 Pathology/Lab Medicine, All India Institute of Medical Sciences, Patna, IND

**Keywords:** diagnosis, estrogen recepter, progestron recepter, her2neu, breast cancer, prognosis

## Abstract

Background

Carcinoma of the breast is one of the most common cancer in females, with preponderance among urban females. The patient's age, tumor size, lymph node status, histological type, histological grade, lymphovascular invasion, hormonal receptor status, human epidermal growth factor receptor 2 (Her2neu) expression, and Ki-67 labeling index for proliferation rate can help determine the appropriate management strategy in these patients. The authors conducted this descriptive retrospective study to assess the association of Ki-67 with clinicopathological parameters in a newly established institute. This may help guide treatment planning in developing countries.

Methodology

Patients diagnosed with primary breast cancer in our institute between January 2017 and March 2020 were included in this study. The clinicopathological prognostic factors were retrieved from the records.

Results

A total of 129 cases of core needle biopsy and mastectomy specimens were included in this study. The patient's mean age and median age were 47.41 and 47 years, respectively. Only 56 specimens of mastectomy were received. T2 (26/56) was the most common tumor size. Grading was done in 46 cases, and grade 2 (23/46) was the most common. Estrogen, progesterone, and Her2neu were positive in 65, 61, and 59 cases, respectively. Only estrogen receptor (ER) expression (p = 0.035) and Her2neu (p = 0.035) overexpression were significantly correlated with Ki-67.

Conclusions

Ki-67 expression was correlated with clinicopathological factors. Only ER expression and Her2neu overexpression were significantly associated with Ki-67. Hence, patients with high Ki-67 expression may have better responses to hormonal therapy and chemotherapy.

## Introduction

Breast carcinoma is the major worldwide cause of cancer-associated death in females [1,2]. In India, breast carcinoma is the most common cancer in females, with a peak age between 50 and 64 years [3]. Urban females in India are more likely to develop breast carcinoma than rural women [3]. There are vast clinical presentation and behavior variations in different patients and racial populations due to genetic heterogeneity [4]. Various clinicopathological factors help evaluate the prognosis and determine the appropriate management strategy in breast cancer patients [3-5]. Factors include patient age, tumor size, lymph node status, histological type, histological grade, lymphovascular invasion, hormonal receptor status, human epidermal growth factor receptor 2 (Her2neu) expression, and Ki-67 labeling index [3-5]. The various molecular techniques are used to ascertain the molecular classification of breast carcinoma [1,2]. The various molecular classification helps determine suitable, specific, and personalized targeted breast cancer treatments [1]. The molecular classification divides breast cancer into five groups: luminal A, luminal B, Her2-enriched, basal-like, and normal breast-like [1,2,5]. The first four groups are major molecular groups that may be assessed using surrogate immunohistochemistry (IHC) markers for estrogen receptor (ER), progesterone receptor (PR), Her2neu, and Ki-67 [1,5]. ER is a steroid receptor transcription factor, especially ER-α, which is primarily used for predicting the response to hormone treatment and has been associated with poor response to chemotherapy [6]. The ER-α expression constitutes the majority of luminal tumors: luminal A and luminal B [6]. However, luminal B has shown increased expression of proliferative markers [6]. PRs are regulated by estrogen-regulated genes and are expressed in >50% of ER-positive breast cancers but are rarely seen in ER-negative cancers [6]. The low PR expression or negative PR in ER-α positive cases is aggressive breast neoplasms associated with poor prognosis [6]. The overexpression/amplification of Her2neu is seen in 15%-30% of patients with breast cancer [2,6]. Although Her2neu positivity is associated with poor prognosis, it predicts response for targeted therapy against Her2neu [2,6]. Ki-67 is a nuclear protein used for the detection and quantiﬁcation of proliferating cells. Its high nuclear expression is associated with a poor prognosis [1,6]. Ki-67 levels have prognostic and predictive roles in breast cancer to predict the chemotherapy response and disease-free survival [6]. Ki-67 also helps differentiate between luminal A and luminal B subtypes, and a cut-off value of 14% has been proposed by many authors [5,6].

The purpose of this descriptive retrospective study was to assess the association of Ki-67 levels with clinicopathological parameters in a convenience sample of adults with breast cancer. Breast cancer is the leading cause of cancer death, and we expect that some clinicopathological parameters of breast cancer patients may have an association with Ki-67. This may help guide treatment planning in developing countries.

## Materials and methods

This study was a descriptive, retrospective study approved by the Institutional Ethical Committee. Eligible sample adult patients had been diagnosed with primary breast cancer in the authors’ institute between January 2017 and March 2020. All the breast cancer cases with both histological and immunohistochemical diagnoses were included in this study. Those patients with metastatic tumor to the breast and undergoing chemotherapy for any malignancy were excluded from this study. The tumor staging was performed following the tumor-node-metastasis (TNM) classification criteria using the 8th Edition of the American Joint Committee on Cancer (AJCC).

After Institutional Ethical Committee approval, data concerning the following clinicopathological prognostic factors were retrieved from patient records: age, tumor size, histological tumor type, Nottingham histological grade system, lymph node involvement, lymphovascular invasion, tumor stage, and IHC results of ER, PR, HER2, and Ki-67. The authors completed the histopathology evaluations using formalin-fixed paraffin-embedded tissue blocks of surgically resected specimens or biopsy tissues. The sections of 3µ to 4µ from the blocks were stained with hematoxylin and eosin (H&E). The IHC was performed according to the VENTANA BenchMark, a fully automated IHC strainer.

The monoclonal antibody was used for ER (Ventana, Rabbit, SP1), PR (Ventana, Rabbit, 1E2), Her2neu (Ventana, Rabbit, 4B5), and Ki-67 (Ventana, Rabbit, 30-9); ≥1% of nuclear immunoreactive tumor cells were considered positive for ER and PR. The Ki-67 was reported in terms of percentage in the hotspot area. As there is no common consensus on a cut-off of Ki-67, the authors considered 10% of Ki-67 as a cut-off value for low-proliferative and high-proliferative tumors. The Her2neu interpretation guidelines were made by the American Society of Clinical Oncology and the College of American Pathologists Clinical Practice Guideline Update 2018.

Due to the lack of in situ hybridization facility and financial constraints, the authors considered equivocal Her2neu results to be positive only for statistical analyses. The categorical observation of frequency of tumor status was compared by chi-square test with Yates correction. A p-value of ≤0.05 was considered as significant. SPSS Version 22 (IBM Corp., Armonk, NY) was used for statistical analysis of the data.

## Results

A total of 129 patients diagnosed with breast carcinoma by core needle biopsies and mastectomy specimens were included in the analytic sample. The age of the patients ranged from 20 to 75 years, with a mean age of 47.41 years and a median age of 47 years, with a standard deviation of 11.36. The most affected age group was 41 to 50 years followed by 31 to 40 years. The most common tumor diagnosis was invasive breast carcinoma - no special type (122/129). Three (2.3%) cases of medullary carcinoma, two (1.5%) lobular carcinoma cases, and one (0.7%) case each of metaplastic carcinoma and mucinous carcinoma were observed. Since most patients had received chemotherapy after their core needle biopsy results, data from only 56 (43.4%) specimens of mastectomy cases were received. In these 56 cases, T2 (26) was the most common and T1 (6) was the least common tumor size category. The lymph node, lymphovascular invasion, and perineural invasion were observed in 33, 27, and 15 cases, respectively. The authors evaluated tumor grade and Nottingham prognostic index scores on 46 mastectomy specimens only. Grade 2 (23) was the most common followed by grade 3 (17) and grade 1 (6) in these 46 cases. ER, PR, and Her2neu were positive in 65, 61, and 59 cases, respectively. A subgroup of 12 cases was equivocal for Her2neu and not evaluated for in situ hybridization due to lack of facility in the authors’ institute. For Ki-67 expression, tumor samples were classified as “low-proliferative” tumors when Ki-67 was ≤10% and “high-proliferative” tumors when Ki-67 was >10%. Ki-67 expression was correlated with clinicopathological factors, and p-value was calculated. Only ER expression (p = 0.035) and Her2Neu overexpression (p = 0.05) were significantly correlated with Ki-67 (Table 1).

**Table 1:** Association of Ki-67 with clinicopathological factors ER, estrogen receptor; Her2neu, human epidermal growth factor receptor 2; NPI, Nottingham prognostic index; PR, progesterone receptor

Clinicopathological factors		Ki-67 positive (>10%)	Ki-67 negative (≤10%)	p-Value
Age (129 cases)	≤47	53 (41.08%)	12 (9.30%)	0.6684
	>47	54 (41.86%)	10 (7.76%)	
Tumor size (56 cases)	T1 ≤ 2 cm	4 (7.14%)	2 (3.57%)	0.9860
	T2 >2 cm ≤ 5 cm	25 (44.64%)	8 (14.29%)	
	T3 > 5 cm	12 (21.43%)	5 (8.93%)	
Lymph node status (56 cases)	N0	16 (28.57%)	7 (12.5%)	0.8215
	N1	8 (14.29%)	3 (5.36%)	
	N2	8 (14.29%)	5 (8.93%)	
	N3	8 (14.29%)	1 (1.79%)	
Lymphovascular invasion (56 cases)	Present	19 (33.93%)	8 (14.29%)	0.6428
	Absent	22 (39.29%)	7 (12.5%)	
Perineural invasion (56 cases)	Present	11 (19.64%)	4 (7.14%)	0.7424
	Absent	30 (53.57%	11 (19.64%)	
Histological grade (46 cases)	Grade 1	4 (8.7%)	2 (4.35%)	0.9441
	Grade 2	18 (39.13%)	5 (10.87%)	
	Grade 3	14 (30.43%)	3 (6.52%)	
NPI (46 cases)	Good prognostic (≤3.4)	3 (6.52%)	2 (4.35%)	0.8521
	Moderate prognostic (3.4-5.4)	19 (41.30%)	4 (8.7%)	
	Poor prognostic (>5.4)	14 (30.43%)	4 (8.7%)	
ER (129 cases)	Positive	50 (38.76%)	15 (11.63%	0.0350
	Negative	58 (44.96%)	6 (4.65%)	
PR (129 cases)	Positive	48 (37.21%)	13 (10.08%)	0.1425
	Negative	60 (46.51%)	8 (6.20%)	
Her2neu (129 cases)	Positive	63 (48.84%)	8 (6.20%)	0.0531
	Negative	44 (34.11)	14 (10.85%)	
Molecular subtypes (129 cases)	Luminal A + B	59 (45.74%)	15 (11.63%)	0.3290
	Her2 enriched	27 (20.93%)	5 (3.86%)	
	Triple negative	22 (17.05%)	1 (0.78%)	

## Discussion

The present study observes that the mean age for breast tumors was 47.14 years, with T2 being the most common tumor size. Our study found that the most common tumor was invasive breast carcinoma (no special type) and the commonest tumor grade was grade 2. We also observe that only ER expression and Her2neu overexpression were significantly correlated with Ki-67. Many authors claimed that tumors that proliferate expeditiously or have a high proliferative index are associated with poor prognosis despite hormonal management or chemotherapy [7]. However, some studies observed no significant difference in outcome between rapidly proliferating tumors and slowly growing tumors after hormonal therapy or chemotherapy [7]. Some studies use a 10% cut-off value of Ki-67 to debar the patient from chemotherapy overtreatment [8]. On the contrary, 25% of Ki-67 is used to cognize chemotherapy susceptible patients [8]. However, there is no general consensus about using the Ki-67 cut-off value for the clinical management of breast cancer [8]. In 2019, Kanyilmaz et al. used low (<10%), intermediate (10-25%), and high (>25%) values of Ki-67 to assess its prognostic weightage [9]. Since most studies use different cut-off values for Ki-67, the results varied for the clinicopathological association. We divided cases into ≤10% or >10% of Ki-67 to examine the association with other clinicopathological parameters similar to the study by Kamranzadeh et al. [8]. Postmenopausal and older females have the best prognosis, and young age may act as an independent risk factor for recurrence and distance metastasis [5]. Older age females have more well-differentiated tumors, while young age females have adverse histologic features [5].

There are many studies to establish the association between clinicopathological factors and the value of Ki-67 (Table 2).

**Table 2:** Comparison of recent studies assessing the association of Ki-67 with other clinicopathological factors BMI, body mass index; ER, estrogen receptor; Her2, human epidermal growth factor receptor 2; PR, progesterone receptor

Author(s)	Year	Ki-67	Not associated/correlated with	Associated/correlated with
Kamranzadeh et al. [8]	2019	≤10 % vs >10%	Menopausal status, ER expression, PR expression, Her2 expression, lymph node status, tumor size, tumor grade	Pathological stage
Kanyilmaz et al. [9]	2019	<10 % vs 10%-25% vs >25% and mean Ki-67 value	Age, menopausal status, tumor stage,	Tumor grade, lymph node status, extracapsular extension, ER expression, PR expression, Her2 expression, lymphovascular invasion
Ragab et al. [10]	2018	<20% vs >20%		Age, tumor grade, tumor size, lymph node status, ER expression, PR expression, Her2 expression
Hashmi et al. [11]	2019	≤15 % vs 15%-24% vs 25%-44% vs >44% in triple-negative breast cancer	Tumor stage, lymph node status, lymphovascular invasion	Age, tumor grade, histological subtypes
Zhu et al. [12]	2020	<30% vs >30%	BMI, location, multifocality, pN	Age, differentiation, pT
Soliman and Yussif [13]	2016	<15% vs >15%	Age, tumor size, lymph node status, tumor stage, histological type, Her2 expression	Tumor grade, mitotic count, ER expression, PR expression, molecular subtype, alive or dead, recurrence and metastasis
Shetty and Rao [14]	2019	Negative vs <10% vs >10% in triple-negative breast cancer	Age, histological type, tumor size, lymph node status	Tumor grade
Thangarajah et al. [15]	2017	<10% vs 10%-20% vs >20%		Tumor grade, tumor stage, lymph node status
Aman et al. [16]	2019	≤15% vs 16%-30% vs >30% and mean Ki-67 value	Mean age, menopausal status, Her2 expression, categories of Ki-67	Histological type, grade, ER, PR (with mean Ki-67 only), molecular subtype
Present study	2020	≤10% vs >10%	Age, tumor size, lymph node status, lymphovascular invasion, perineural invasion, histological grade, Nottingham prognostic index, PR expression, molecular subtypes	ER expression, Her2 expression

The differences in the compared studies may be due to different study designs, study populations, different tumor types, and using different Ki-67 cut-off values.

Ragab et al., Hashmi et al., and Zhu et al. observed the association between age and Ki-67 [10-12]. In contrast, Kanyilmaz et al., Soliman and Yussif, and Shetty and Rao did not find this association as observed found in our study [9,13,14]. This could be due to different median ages and cut-off values of Ki-67. The tumor size correlates with the incidence of lymph node metastases and survival rate, which is not followed by basal-like breast carcinomas [5]. The tumor size strongly predicts the dissemination and rate of relapse in lymph node-negative breast carcinomas [5]. We observed that the tumor size was not associated with the Ki-67 value, which is concurrent with the findings of Kamranzadeh et al., Zhu et al., Soliman and Yussif, and Shetty and Rao [8,12-14].

One of the most important prognostic parameters is axillary lymph node metastases [5]. The survival rate depends on the number of involved lymph nodes, the level of the involved nodes, and the absence or presence of extranodal extension [5]. The lymph node status was also not associated with the Ki-67 in our study, as also observed by Kamranzadeh et al., Soliman and Yussif, Shetty and Rao, and Hashmi et al. [8,11,13,14]. This is contrary to the observation of Kanyilmaz et al., Ragab et al., and Thangarajah et al [9,10,15]. The risk of distant recurrence is allied with lymphatic tumor emboli [5]. Similar to Hashmi et al.’s finding, the lymphovascular invasion was not associated with Ki-67 in our study, which contradicts the finding by Kanyilmaz et al. [9,11]. The tumor grade is directly correlated with the prognosis [5]. Many studies have found a correlation between tumor grade and Ki-67. Our study observed no association between tumor grade and Ki-67, which is concurrent with the finding of Kamranzadeh et al. [8].

The present study has the limitation of a lesser number of cases with available tumor size, lymph node status, lymphovascular invasion, and tumor grade, along with different cut-off Ki-67 values. ER expression, PR expression, and Her2 expression also do not have a common consensus in the literature compared with the expression of Ki-67. Kanyilmaz et al., Ragab et al., Soliman and Yussif, and Aman et al. observed the association of hormonal expression with Ki-67 [9,10,13,16]. In contrast, Kamranzadeh et al. failed to find this association [8]. The overexpression or amplification of Her2neu predicts the response to Her2-targeted therapies [5]. Kanyilmaz et al. and Ragab et al. found the association of Her2 with Ki-67, which is the opposite of Kamranzadeh et al., Soliman and Yussif, and Aman et al. [8-10,13,16]. In our study, ER expression and Her2 expression were associated with Ki-67, while PR expression was not.

Contrary to Soliman and Yussif and Aman et al., our study did not observe molecular subtyping association with Ki-67 [13,16]. Here we also state that equivocal Her2 cases were not evaluated by in situ hybridization.

In our opinion, the association between Ki-67 and other clinicopathological factors varies in the literature may be due to non-uniformity for the cut-off value of Ki-67.

Study limitations

Our study was conducted at a single cancer center in India with a small sample size. Another limitation was the lack of facility of in situ hybridization for equivocal Her2 evaluation. Large-scale studies from the same sample population are required to strengthen our observations. This may help predict the hormonal and chemotherapy response in breast cancer patients in the financial constraint population using Ki-67. Also, the uniformity for the cut-off value of Ki-67 is necessary to make observations generalizable.

## Conclusions

Based on these results, we conclude that Ki-67 expression is significantly associated with ER expression and Her2neu overexpression in breast cancer patients who received care in our institute. We observed a high proliferative rate in ER-positive and Her2neu positive cases, which is an important prognostic marker. We did not observe a significant correlation with other clinicopathological parameters included in the study. We also require uniformity for the cut-off value of Ki-67 to make observations generalizable.
